# Meningeal carcinomatosis: three case-reports

**DOI:** 10.1186/s12957-018-1376-8

**Published:** 2018-04-13

**Authors:** Guangyong Chen, Long Ma, Meiling Xu, Xuemei Wang, Chong Wang, Conghai Zhao, Jinnan Zhang

**Affiliations:** 1 Department of Neurosurgery, China-Japan Union Hospital of Jilin University, Changchun, 130033 China; 2 Department of Rheumatology, China-Japan Union Hospital of Jilin University, Changchun, 130033 China; 3 Department of Pathology, China-Japan Union Hospital of Jilin University, Changchun, 130033 China

**Keywords:** Meningeal carcinomatosis, Cerebrospinal abbreviation cytology, MRI, Enhanced MRI

## Abstract

**Background:**

Meningeal carcinomatosis (MC) is characterized by diffuse infiltration of tumor cells in meninges. There is no tumor mass in the brain and parenchyma of the spinal cord. MC is divided into primary and metastatic types. MC cases were previously diagnosed postoperatively or at autopsy. Recent advances in spinal abbreviation cytology and imaging have led to increase in number of reported cases. In this study, we discuss the manifestations of MC patients based on magnetic resonance imaging (MRI) findings, as well as the correlation between the manifestations and pathology.

**Case presentation:**

MC was confirmed in all three cases by lumbar puncture and gadopentetate dimeglumine-enhanced magnetic resonance imaging. Due to different primary diseases, the patients had specific imaging manifestations.

**Conclusion:**

Enhanced MRI examination is extremely sensitive for detecting abnormalities in meninges, which plays a very important role in the diagnosis of MC. Since meninges of some MC patients cannot be enhanced, the enhanced MRI examination cannot be replaced by conventional cerebrospinal abbreviation examination. Attribute to the diversity of MR contrast agents, which could provide higher lesion conspicuity and enhances lesion detection, there may be some more choices to improve the detection rate of MC patients and prolong their survival lifetime.

## Background

Meningeal carcinomatosis (MC) is an aseptic inflammatory reaction caused by diffuse infiltration of tumor cells from other organs in cerebral pia mater. MC is also called leptomeningeal carcinomatosis (LMC) or carcinomatous meningitis. The prognosis of MC remains poor. The detection rate of MC is increasing with the utilization of developed clinical examination level and improved therapy, but the survival time is increasing. The prognosis of MC mainly depends on the diagnosis and positive treatment of patients in the early stages of disease. However, early-stage diagnosis is often difficult. Previous diagnosis mainly depends on the results of cerebrospinal abbreviation cytology in finding tumor cells. It has been reported that the positive rate of tumor cells during the first lumbar puncture was low. Although multiple lumbar punctures can increase the positive rate, it easily delays the therapy and causes discomfort to the patients [1].

Imaging examination is helpful in diagnosing the disease, especially enhanced MR examination, which demonstrates patient-specific performance. In this study, we retrospectively analyzed the clinical characteristics of three MC patients admitted in our department and summarized their results, clinical manifestations, and imaging manifestations of MC. These consequently provided relevant knowledge for the diagnosis and treatment of MC clinically [2].

## Case presentation

### Case 1

Female, 40 years old, visited our hospital because of discontinuous hypogastralgia for 4 months. After auxiliary examination, the patient was diagnosed with malignant tumor of pelvic cavity and phase II cervical cancer. After four treatment courses of neoadjuvant chemotherapy, the patient received extended total hysterectomy, bilateral adnexectomy, and partial greater omentum resection under venous inhalation anesthesia on December 8, 2016. Postoperative pathological report showed no cancer cells in the peritoneal fluid. In the left adnexal region, the ovary and muscular layer of the fallopian tube were tested. Results showed poorly differentiated adenocarcinoma in the serosa and partial signet-ring cell carcinoma, suggesting the exclusion of metastasis clinically. In the total uterus, more tumor thrombi were observed in the endometrial and muscular layers, cervix uteri, and vessels of vaginal cuff, while the parametrium was not involved. In the greater omentum, poorly differentiated adenocarcinoma invasion was observed in the adipose tissue of the omentum. In the right adnexal region, the ovary and muscular layer of the fallopian tube were tested, which showed poorly differentiated adenocarcinoma in the serosa and partial signet-ring cell carcinoma. A9 immunohistochemistry revealed CK7(+) CK20(+) Villin (+) CDX-2(+) P16(+) CA125(−) ER (−) PR (−) EGFR (weak+) P53(−) Ki67(70%+) Cadherin-17(+). After surgery, the patient received two courses of treatment, and the chemoradiotherapy regimen was as follows: paclitaxel of 240 mg, carboplatin of 300 mg, and simultaneous treatment was given to stop vomiting and for liver protection. No side effects were observed by significant chemotherapy (such as nausea and vomiting). Two weeks after chemotherapy, the patient had symptoms such as headache, nausea, and vomiting, and the symptoms were significantly aggravated at 3 days after hospitalization in the Neurosurgery Department. The patient was then transferred to our department as metastatic cancer has been diagnosed by the Outpatient Department. Diet and sleep conditions remained poor during the disease course but had no abnormal fecal and urinary functions. No significant changes in the body weight were observed. Results of cerebrospinal abbreviation examination performed on February 27, 2017, and March 01, 2017, were shown in Table 1.

**Table 1:** Results of cerebrospinal fluid examination performed on February 27, 2017, and March 01, 2017

	Color	Transparency	Pandy’s reaction	Leukocyte count (10~ 6/L)	Sugar (mmol/L)	Protein (g/L)	Chloride (mmol/L)	Classification of leukocyte (mononuclear) (multinuclear)
First	No color	Transparent	(−)	61	2.0	0.53	126.2	93%, 7%
Second	No color	Transparent	(−)	36	2.6	0.44	127.1	97%, 3%

**Fig. 1:**
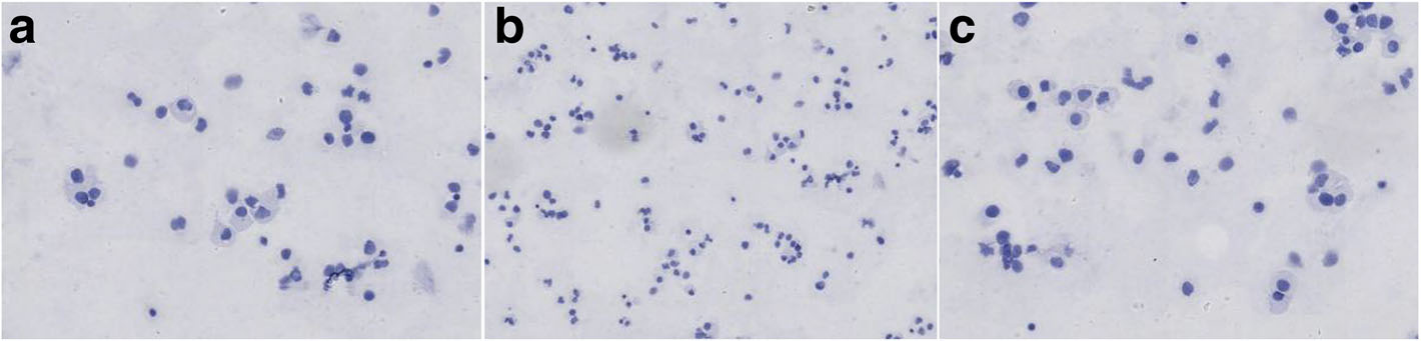
Examination of shedding cells in the cerebrospinal abbreviation in **a** and **b** and **c** specimens

Cerebrospinal abbreviation findings indicated that the size of the tumor cells was abnormal, the hyperchromatic karyosome distribution was nonuniform, cells were crowded without polarity, and the karyotheca was thickened and rough and dispersed in the lymphocytes (Fig. 1).

**Fig. 2:**
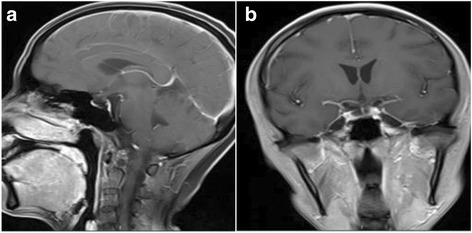
Abnormal enhancement shadow was observed in the tentorium cerebelli and basicranial sella region in **a** and **b** MRI images

MRI indicated abnormal strip-shaped high-density shadow on the top and occipitalia regions (Fig. 2).

**Fig. 3:**
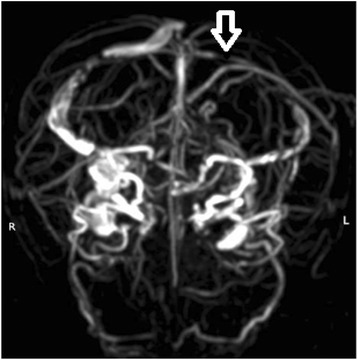
Right transverse sinus shadow showed continuous break-off and hindered venous sinus reflux

Magnetic resonance venography (MRV) imaging examination showed unclear left transverse sinus, clear imaging of sinus sagittalis superior, sinus sagittalis inferior, great cerebral veins, sinus confluens, sinuses rectus sinus sigmoideus, and right transverse sinus with normally arranged vessels. There were no significant filling defects (Fig. 3). Radiotherapy and chemotherapy were recommended, while the patient chose whole-brain radiotherapy and died 4 weeks later.

### Case 2

Male, 64 years old, showed symptoms like dizziness and headache with nausea 1 month before hospitalization. Headache was manifested as a whole-brain discontinuous distending pain. The pain was not significantly relieved even after oral administration of painkillers. Headache was gradually aggravated for the past week before hospitalization, and so, the patient visited our hospital. Head MRI diagnosed intracranial space-occupying lesion, and hence, the patient was hospitalized. During the disease course, the patient had no disturbances of consciousness and fever but had frequent vomiting and cramps, as well as poor sleep and diet conditions. The patient underwent lower right lung cancer radical operation 3 months ago, and the pathological report indicated squamous cell lung carcinoma.

The results of cerebrospinal abbreviation on July 05, 2016, were shown in Table 2.

**Table 2:** The results of cerebrospinal fluid on July 05, 2016

	Color	Transparency	Pandy’s reaction	Leukocyte count (10~ 6/L)	Sugar (mmol/L)	Protein (g/L)	Chloride (mmol/L)	Classification of leukocyte (mononuclear) (multinuclear)
Cerebrospinal fluid	No color	Transparent	(±)	13	1.1	0.81	123.7	94%, 6%

**Fig. 4:**
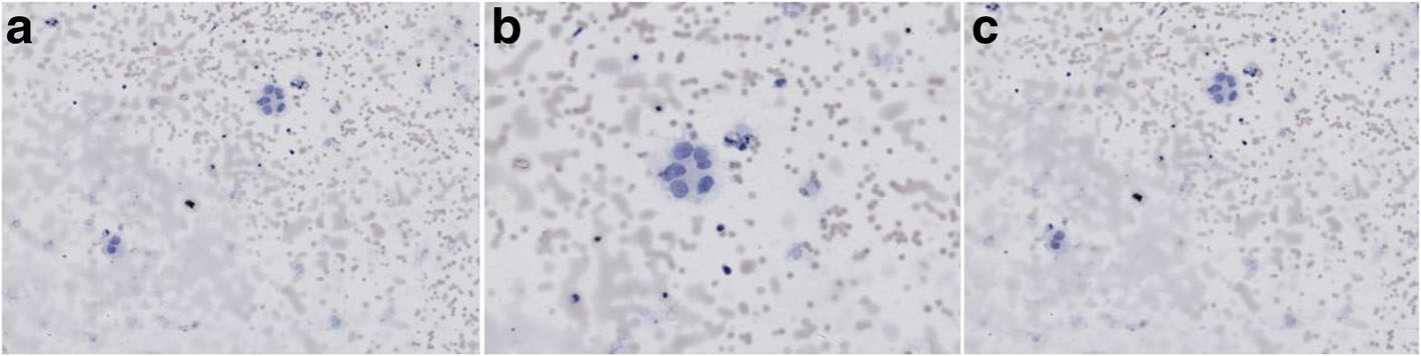
Examination of shedding cells in the cerebrospinal abbreviation in **a** and **b** and **c** specimens

Cerebrospinal abbreviation findings of tumor cells indicated (Fig. 4) dispersion of cerebrospinal abbreviation in the heterocysts, cells were large, and the cytoplasm was rich and basophilic. Cell nucleus appeared circular, and the chromatin was exquisite where small lymphocytes were observed.

**Fig. 5:**
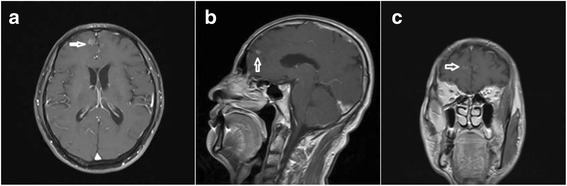
Arrow indicates very clear enhanced shadow in **a** and **b** and **c** MRI images

Enhanced MR scan indicated patchy abnormal enhancement shadow in the right frontal lobe with irregular margin (Fig. 5). This patient chose chemotherapy and died 6 weeks later.

### Case 3

Female, 54 years old, who had a clear history of lung cancer was diagnosed with moderately differentiated adenocarcinoma on November 12, 2013, in Peking Union Medical College Hospital. Pathological changes showed a large amount of micropapillary adjacent to the tunica. EGFR Exon19 deletion mutations were detected. When the patient was hospitalized on August 13, 2014, multi-bone metastases, metastatic carcinoma to the spleen, multiple metastatic carcinoma to the liver, and urinary tract infection were seen. The patient received chemotherapy cycles in the Hematology Department. The onset of the disease was initiated by headache and nausea.

**Fig. 6:**
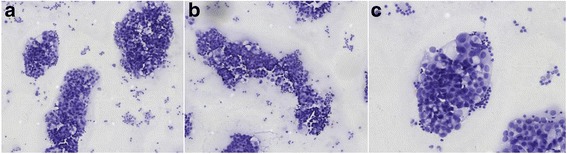
Examination of shedding cells in the cerebrospinal abbreviation in **a** and **b** and **c** specimens

Cerebrospinal abbreviation findings of tumor cells indicated (Fig. 6) cluster arrangement with circular shape and in large volume. The cytoplasm was rich in fine granules, slightly basophilic and translucent, with a few vacuolus. The nuclear egg was circular and off-normal. The nuclear membrane was obvious, and the karyosome was uniformly distributed in the form of fine granules. Inclusion bodies were clearly observed in the nucleus.

**Fig. 7:**
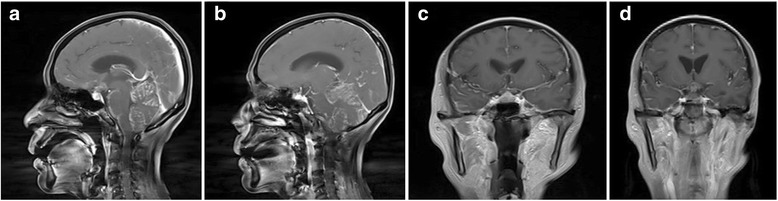
Abnormal enhancement shadow near tentorium cerebelli seen in **a**, **b**, **c**, and **d**

Strip-shape enhancement of cerebellum and occipital sulus observed by enhanced MR scan (Fig. 7). This patient was treated with Gefitinib tablet and died 4 months later.

## Discussion and conclusions

The gold diagnosis standard is CSF cytology with the specificity of about 95% for MC [3]. However, nearly 50% of patients are cytologically negative on the first cerebrospinal abbreviation examination. The lesions can be observed in the cerebrospinal abbreviation when they diffuse into the ventricular system or meninges. However, the tumor cells in the cerebrospinal abbreviation were observed after repeat examinations [4, 5].

Most of the MC patients do not suffer from brain metastasis. The tumor cells mainly diffuse and infiltrate into the meninges or subarachnoid space, without significant space-occupying effect. Furthermore, there was no significant difference between the pathological tissue and adjacent cerebrospinal abbreviation [6]. The disease is often initiated by headache. The authors believed that the disease was caused by the pressure of metastasis on the meninges to stimulate local sensory nerve. Meanwhile, in the MRV examination of case 1, we observed that the left transverse sinus was unclear. This is because the patient had cancer, and the blood was in hypercoagulable state. The patient had headache, which might be related to sinus thrombosis. Meanwhile, the possibility of venous sinus endothelial metastasis of tumor was not excluded. However, there was no any report that takes MRV as the conventional examinations in MC patients.

In terms of enhanced MR, the differences were as follows: (1) lung cancer metastasis occurred in two cases, and their imaging examinations were similar to a certain level. Under enhanced MR, enhancement of the pachymeninges showed continuous enhancement, which was diffuse reinforcement, whereas another patient with intracranial metastasis of ovarian cancer had discontinuous enhanced MR, showing nodular enhancement [7]. Most of the lung cancer metastasis shows diffuse reinforcement in the blood channels, while lymphatic metastasis and abdominal cavity implantation metastasis remain the main reasons for ovarian cancer. The author believed that the relevance between MC imaging manifestation and metastasis and growth of tumor cells was that the tumor cells often grew in diffuse and focal way. Diffuse growth spreads along with the surface of meninges, involving endocranium, and spreads along with intracranial flank, cerebral falx, and tentorium cerebelli. When pia mater and subarachnoid space were involved, it often stretches into the sulus. The focal growth can occur alone or by combining with diffuse growth, which compels tumor cell aggregation in the surface of meninges, subarachnoid space, or ependyma, forming different-sized focal nodules. Thus, the former one manifests as diffuse linear enhancement and the latter as nodular enhancement. Through enhanced MR, we deduced that most of the MC that metastasizes by blood channels show diffuse enhancement. The MC metastasized by lymph and implantation showed nodular enhancement. (2) Different sites include MC metastasized by blood channel mostly occurs under the base of the skull or in the sites with rich blood supply, and the MC metastasized by lymph might be the sites with poor blood supply.

Manifestations of MC in PET-CT: the abnormal appearance of MC in PET-CT may be seen earlier than enhanced MR. For the patients with negative partial enhanced MR, the abnormity can be found in PET-CT. However, we have not seen much related reports so far, and according to the cost of PET-CT, it cannot be widely used and carried out in Chinese clinical practice [8, 9].

We all used gadopentetate dimeglumine as MR contrast agents for these three cases in our hospital. According to a recent study, Pan et al. [10] reported that gadobutrol provides higher lesion conspicuity and enhances lesion detection in meningeal metastasis compared with gadopentetate dimeglumine in two MC patients, which is a new extracellular contrast agent that belongs to non-ionic macrocyclic gadolinium chelate. The lower osmolality and viscosity of gadobutrol enables the double-concentrated solution, which contains twice the amount of Gd chelate per volume. The T1 relaxivity of gadobutrol is approximately 14–27% higher than that of other 0.5-mol/L Gd chelates [11], such as gadopentetate dimeglumine. Therefore, gadobutrol-enhanced MR may be a better choice that facilitates early diagnosis of MC; meanwhile, its clinical value has to be verified with additional clinical studies.

Above all, enhanced MR examination is extremely sensitive to the abnormity of meninges, which plays a very important role in the diagnosis of MC. Because meninges of few MC patients cannot be enhanced, the enhanced MR examination cannot be replaced by conventional cerebrospinal abbreviation examination. Attribute to the diversity of MR contrast agents, which could provide higher lesion conspicuity and enhance lesion detection, there may be some more choices to improve the detection rate of MC patients and prolong their survival lifetime.

## Abbreviations

LMC: Leptomeningeal carcinomatosis
MC: Meningeal carcinomatosis
MRV: Magnetic resonance venography
